# Disappearance of local ventricular components during extrastimulus pacing after ultra-low-temperature cryoablation for ventricular tachycardia: a case report

**DOI:** 10.1093/ehjcr/ytag721

**Published:** 2026-09-23

**Authors:** Kohei Ukita, Roman Mamaev, Tuğba Aktemur Özalp, Jan-Per Wenzel, Roland Richard Tilz

**Affiliations:** Department of Rhythmology, University Heart Center, University Hospital Schleswig-Holstein, Lübeck, Germany; Department of Rhythmology, University Heart Center, University Hospital Schleswig-Holstein, Lübeck, Germany; Department of Rhythmology, University Heart Center, University Hospital Schleswig-Holstein, Lübeck, Germany; Department of Rhythmology, University Heart Center, University Hospital Schleswig-Holstein, Lübeck, Germany; German Center for Cardiovascular Research (DZHK), Partner Site Hamburg/Kiel/Lübeck, Lübeck, Germany; Department of Rhythmology, University Heart Center, University Hospital Schleswig-Holstein, Lübeck, Germany; German Center for Cardiovascular Research (DZHK), Partner Site Hamburg/Kiel/Lübeck, Lübeck, Germany

**Keywords:** Case report, Catheter ablation, Cardiac resynchronization therapy defibrillator, Left ventricular outflow tract, Ultra-low-temperature cryoablation, Ventricular tachycardia

## Abstract

**Background:**

Ultra-low-temperature cryoablation (ULTC) enables the creation of deep and potentially transmural myocardial lesions and is increasingly used for ventricular tachycardia (VT) substrate modification. However, the chronic electrophysiological properties of myocardium following extreme cryothermal injury remain incompletely understood.

**Case summary:**

A 66-year-old man with dilated cardiomyopathy and a cardiac resynchronization therapy defibrillator presented with recurrent sustained VT 15 months after ULTC of the left ventricular outflow tract. During repeat ablation, high-density mapping revealed sharp local ventricular electrograms within the prior ablation region. Programmed ventricular stimulation from the right ventricular outflow tract reproducibly induced disappearance of these sharp local components during extrastimulus pacing, while the surface QRS complex remained preserved, indicating preserved global ventricular activation. The phenomenon was consistently observed across different pacing protocols. Radiofrequency ablation targeting this region resulted in elimination of the sharp local electrograms, whereas dull far-field signals persisted. After ablation, VT was no longer inducible, and the patient remained free from recurrence at 3-month follow-up.

**Discussion:**

This case demonstrates a reproducible disappearance of near-field ventricular electrograms during extrastimulus pacing in myocardium previously treated with ULTC. The findings suggest localized refractoriness or source–sink mismatch within partially injured myocardium, rather than conventional slow conduction. These observations provide novel insight into the chronic electrophysiological effects that may occur after ULTC and may have implications for substrate assessment during repeat VT ablation.

Learning points

- Disappearance of local ventricular electrograms during extrastimulus pacing was observed after ultra-low-temperature cryoablation for ventricular tachycardia.
- Abrupt electrogram disappearance may reflect functional conduction failure rather than slow conduction.
- Repeat high-density mapping may identify residual arrhythmogenic substrate after ultra-low-temperature cryoablation.

## Introduction

Ultra-low-temperature cryoablation (ULTC) is increasingly used to treat ventricular tachycardia (VT) substrates within thick ventricular myocardium by creating deep, contiguous, and potentially transmural myocardial lesions.^1,2^ However, the chronic electrophysiological characteristics of myocardium exposed to extreme cryothermal injury remain unknown. In this report, we describe a case of repeat ablation for VT recurrence performed 15 months after ULTC, in which a reproducible disappearance of local ventricular components was observed during programmed ventricular stimulation despite preservation of the surface QRS complex.

## Summary figure

**Figure:**
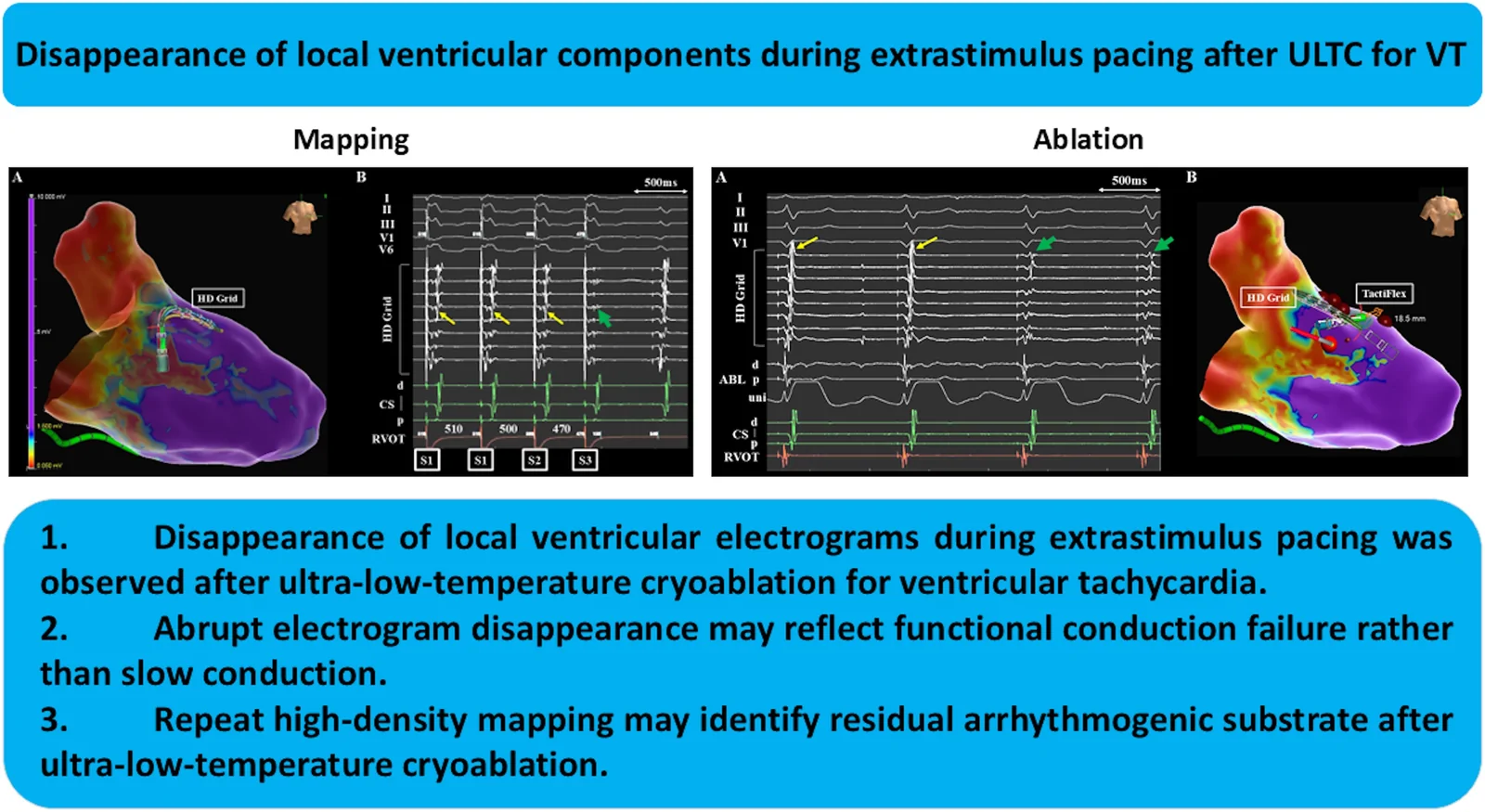


## Case report

A 66-year-old man with dilated cardiomyopathy (left ventricular ejection fraction: 37%) and a cardiac resynchronization therapy defibrillator (CRT-D) underwent repeat ablation for recurrent symptomatic sustained VT 15 months after ULTC of the anterior left ventricular outflow tract (LVOT) (−146 to −150°C, four applications, *Figure 1A*). ULTC was selected during the initial procedure because the arrhythmogenic substrate was suspected to be located intramurally in this region. The VT documented at the repeat procedure exhibited an identical QRS morphology to that observed during the initial ablation, with an inferior axis and positive QRS complexes in leads V3-V5 (*Figure 1B*), suggesting recurrence of VT from the same site of origin.

**Figure 1:**
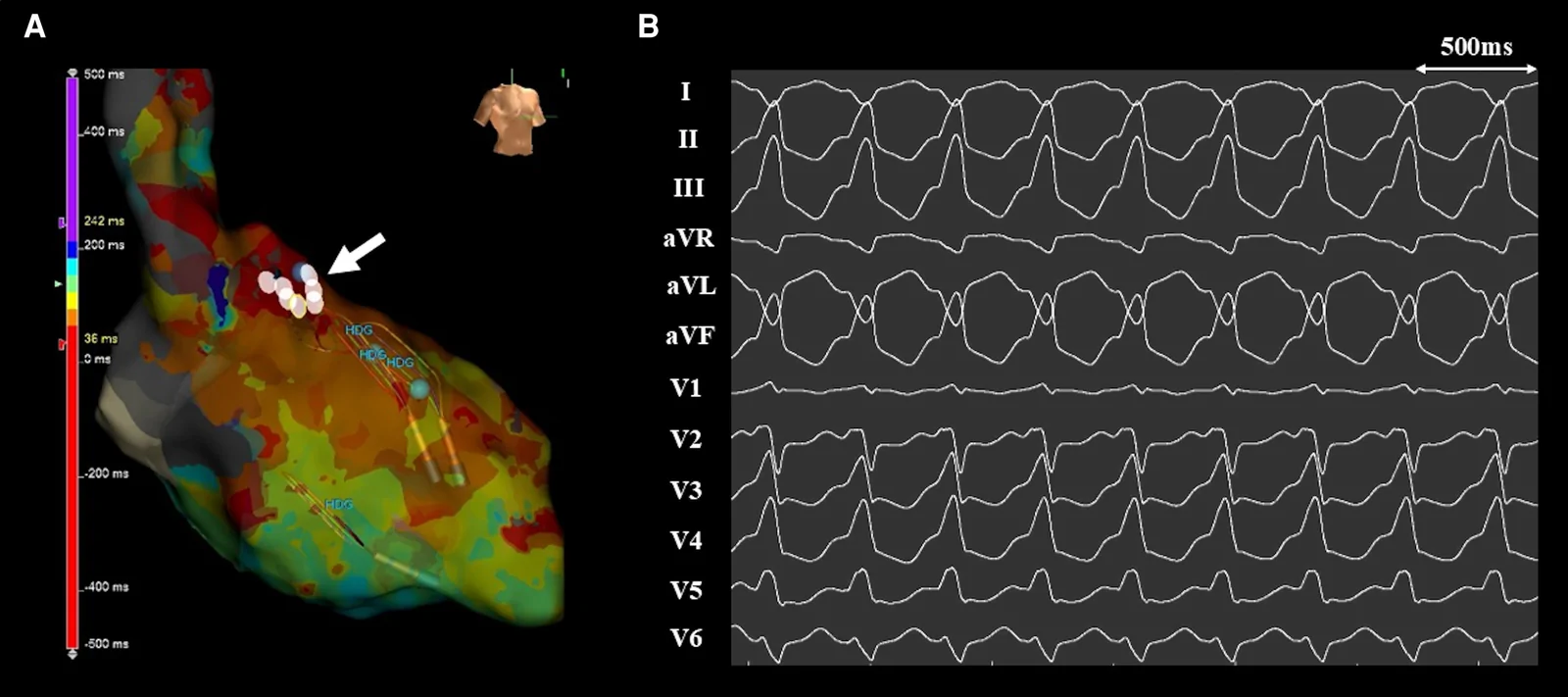
(*A*) Three-dimensional map obtained during the initial ultra-low-temperature cryoablation procedure showing the application sites (white tags indicated by the white arrow) at the anterior left ventricular outflow tract (30° right anterior oblique view). (*B*) Twelve-lead electrocardiogram during ventricular tachycardia. The tachycardia cycle length was 380 ms. The QRS morphology showed an inferior axis and positive in leads V3-V5.

The patient underwent the repeat ablation procedure. The procedure was performed under deep sedation. After transseptal access to the left ventricle (LV), a substrate map was constructed using a multipolar mapping catheter (Advisor^TM^ HD Grid, Abbott). Detailed electrogram assessment was performed to characterize local ventricular signals at the prior ULTC region. With the HD Grid positioned at the LVOT (*Figure 2A*), where the prior ULTC had been performed, extrastimulus pacing was delivered from the right ventricular outflow tract (RVOT). During the stimulation protocol (S1-S1 = 510 ms, S1-S2 = 500 ms, and S2-S3 = 470 ms), sharp local ventricular components recorded by the HD Grid disappeared reproducibly in response to the S3 stimulus, while the surface QRS complex persisted (*Figure 2B*). This phenomenon was also observed during other pacing protocols ([S1-S1, S1-S2, S2-S3] = [510 ms, 500 ms, 460 ms] and [510 ms, 500 ms, 450 ms]). Clinical VT was induced by programmed ventricular stimulation from the RVOT but required termination with electrical cardioversion due to haemodynamic instability. Subsequently, radiofrequency ablation was performed via a transaortic approach using a contact force-sensing catheter (TactiFlex^TM^, Abbott; 30 W, 20–30 g). The sharp ventricular components were eliminated after ablation, while the relatively dull potentials—contained within the QRS complex—remained unchanged (*Figure 3A* and *B*). Following ablation, the clinical VT was no longer inducible with aggressive programmed stimulation.

**Figure 2:**
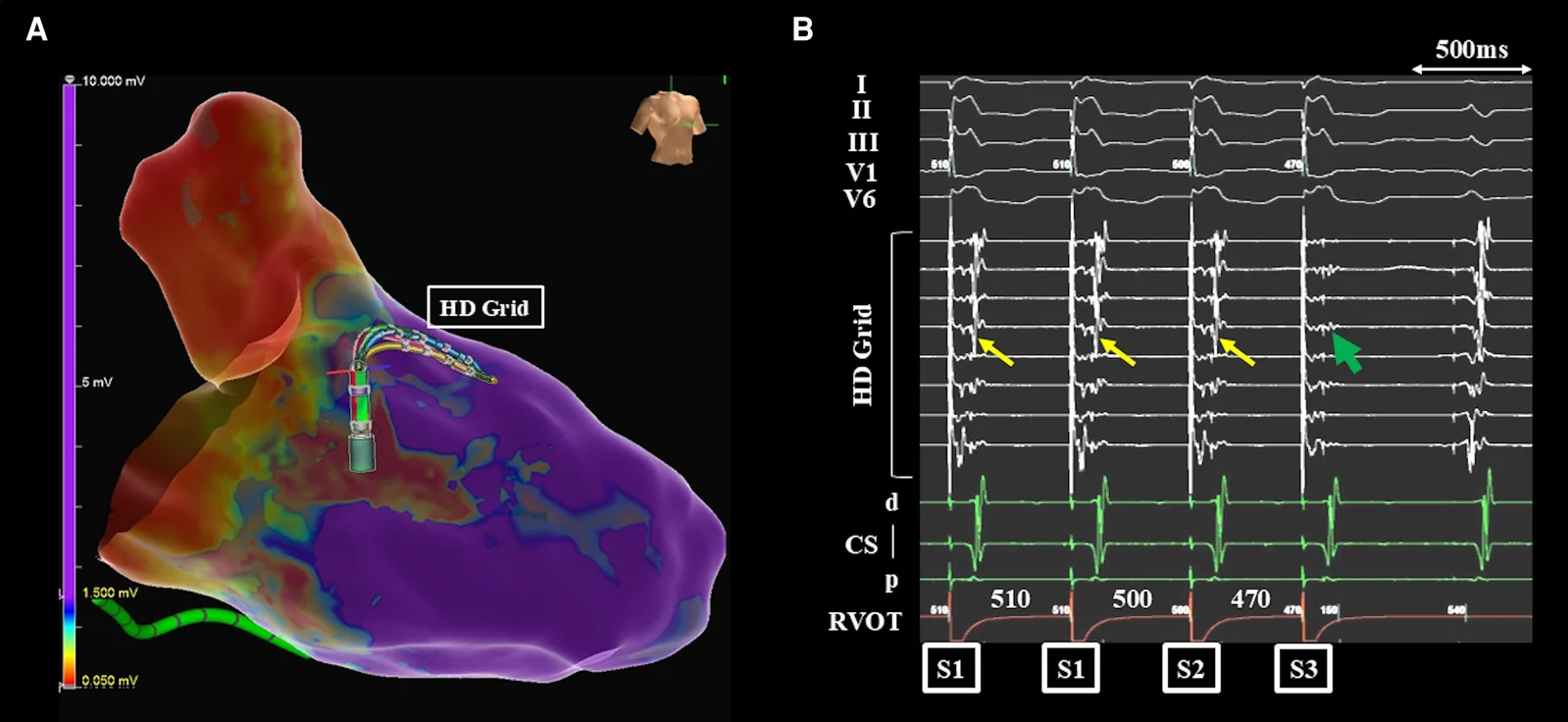
(*A*) Three-dimensional map obtained during the repeat procedure demonstrating the HD Grid catheter positioned at the left ventricular outflow tract (30° right anterior oblique view). (*B*) Intracardiac electrograms during programmed ventricular stimulation from the right ventricular outflow tract. The sharp ventricular electrograms disappear during the S3 extrastimulus (yellow arrows), whereas the dull ventricular electrograms remain preserved (green arrow). CS, coronary sinus; RVOT, right ventricular outflow tract.

**Figure 3:**
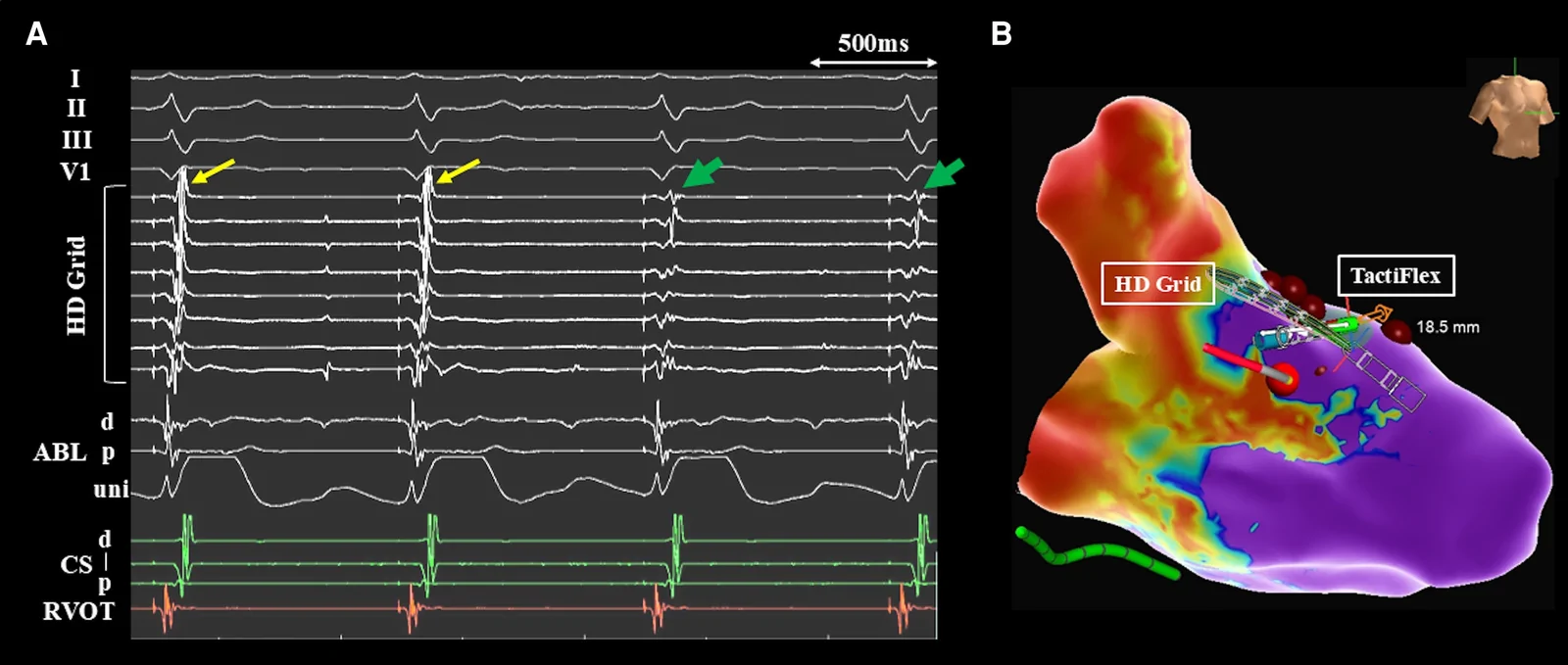
(*A*) Intracardiac electrograms during radiofrequency ablation. The sharp ventricular electrograms are eliminated (yellow arrows), whereas the dull ventricular signals remain unchanged (green arrows). (*B*) Three-dimensional map demonstrating the positions of the HD Grid and TactiFlex at the time of elimination of sharp ventricular components on the HD Grid (30° right anterior oblique view). ABL, ablation catheter; CS, coronary sinus; RVOT, right ventricular outflow tract.

No complications occurred during the procedure. At the 3-month follow-up, no recurrence of sustained VT was documented in the CRT-D and the patient was asymptomatic (*Table 1*).

**Table 1:** Timeline of events

Day 0	Repeat ablation procedure for sustained VT was performed
Day 2	The patient was discharged without complications
Day 92	No recurrence of sustained VT was documented in the CRT-D and the patient was asymptomatic

CRT-D, cardiac resynchronization therapy defibrillator; VT, ventricular tachycardia.

The patient provided written informed consent for the ablation procedure and agreed to the publication of his case details and images in this report.

## Discussion

This case illustrates a reproducible disappearance of sharp local ventricular components during programmed ventricular stimulation despite preserved surface QRS morphology and global ventricular capture. Using high-density grid mapping, the sharp components could be clearly distinguished from dull ventricular signals and were acutely eliminated by focal radiofrequency ablation, suggesting near-field potentials. The residual electrogram activity during the extrastimulus pacing consisted of low-amplitude, broader signals coincident with the surface QRS complex, consistent with far-field ventricular activation. Although localized refractoriness or source–sink mismatch within partially injured myocardium represents one plausible explanation,^3^ alternative mechanisms should also be considered.

First, conventional slow or decremental conduction within diseased myocardium remains a possible explanation. Fractionated electrograms and delayed activation are well-recognized features of ventricular scar and may exhibit conduction abnormalities during premature stimulation.^4^ However, in the present case, the sharp local electrograms disappeared abruptly without preceding progressive delay, increasing fractionation, or temporal dispersion. This behaviour differs from the typical electrogram changes expected during decremental slow conduction and therefore suggests that functional conduction failure, rather than progressive conduction slowing, may have occurred.

Second, the observed findings may simply reflect conduction abnormalities within chronically diseased myocardium unrelated to the previous ULTC lesion. Although the abnormal electrograms were recorded within the region previously treated by ULTC and were abolished by focal radiofrequency ablation, the patient had underlying dilated cardiomyopathy, which itself may produce heterogeneous myocardial fibrosis and functional conduction abnormalities.^5^ Therefore, a direct causal relationship between ULTC and the observed electrogram behaviour cannot be established.

Third, technical factors such as intermittent catheter contact or recording artefacts were carefully considered. These explanations appear less likely because the broader far-field ventricular electrograms remained unchanged throughout the pacing protocol, the catheter position remained stable fluoroscopically, and the phenomenon was reproducibly observed using multiple pacing protocols with different coupling intervals.

Although causality cannot be proven, the observation remains noteworthy because it occurred reproducibly within the previous ULTC lesion and disappeared immediately after focal radiofrequency ablation. This temporal and spatial relationship suggests that localized electrophysiological alterations within the previously treated substrate may have contributed to the phenomenon.

ULTC has been developed to create deeper and more homogeneous lesions than conventional radiofrequency ablation and is increasingly used for ventricular substrate modification.^1,2^ However, despite its ability to create extensive myocardial injury, lesion formation within thick ventricular myocardium may still be spatially heterogeneous. Residual viable myocardial bundles within or adjacent to cryothermal lesions may exhibit heterogeneous electrophysiological properties, including altered excitability and refractoriness, potentially resulting in functional conduction block during ventricular stimulation.

Whether this mechanism is unique to ULTC remains unknown. Similar electrophysiological behaviour may occur in other forms of chronic myocardial disease or following different energy sources used for ventricular ablation. Therefore, rather than demonstrating a ULTC-specific phenomenon, this case may illustrate one potential manifestation of chronic heterogeneous myocardial injury after extensive ventricular substrate modification. Further studies involving larger numbers of patients undergoing repeat mapping after ULTC are required to determine the prevalence and mechanistic basis of these findings.

Several limitations should be acknowledged. First, this is a single observational case, precluding definitive mechanistic conclusions. Second, because the induced VT was haemodynamically unstable, detailed activation mapping and local electrogram recordings during tachycardia were not feasible. Consequently, the relationship between the observed electrogram behaviour and the VT circuit could not be established directly. Third, although the phenomenon occurred within the previous ULTC lesion, the contribution of the underlying cardiomyopathic substrate cannot be excluded. Finally, pathological confirmation of the underlying tissue characteristics was not available.

## Conclusions

This case describes a reproducible disappearance of local ventricular electrograms during extrastimulus pacing in myocardium previously treated with ULTC despite preserved global ventricular activation on the surface QRS complex. These findings suggest localized functional conduction failure, possibly related to heterogeneous chronic myocardial injury, while alternative explanations including conventional slow conduction and intrinsic diseased myocardium cannot be excluded. Further studies are required to clarify the long-term electrophysiological characteristics of myocardium following ULTC and to determine whether similar findings occur after other ventricular ablation technologies.

## Lead author biography


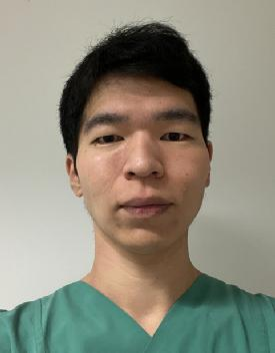


Dr. Kohei Ukita graduated from Osaka University in 2015. After completing his residency, he worked at Osaka Rosai Hospital. Since 2025, he is an Electrophysiology Fellow at the University Hospital Schleswig-Holstein.

## Data Availability

No new data were generated or analysed in support of this paper.

## References

[1] Verma A, Essebag V, Neuzil P, Dyrda K, Balt J, Dinov B, et al Cryocure-VT: the safety and effectiveness of ultra-low-temperature cryoablation of monomorphic ventricular tachycardia in patients with ischaemic and non-ischaemic cardiomyopathies. Europace 2024;26:euae076. 10.1093/europace/euae076 PMC10998960 38582974

[2] Weiss JP, Reddy VY, Tandri H, Richardson TD, Gerstenfeld EP, Stevenson WG, et al Ultra-low cryoablation for scar-related VT ablation: results from the US early feasibility study. Circ Arrhythm Electrophysiol 2026;19:e014095. 10.1161/CIRCEP.125.014095 41623095

[3] Ciaccio EJ, Coromilas J, Wit AL, Peters NS, Garan H. Source-sink mismatch causing functional conduction block in re-entrant ventricular tachycardia. JACC Clin Electrophysiol 2018;4:1–16. 10.1016/j.jacep.2017.08.019 PMC5874259 29600773

[4] Stevenson WG, Weiss JN, Wiener I, Rivitz SM, Nademanee K, Klitzner T, et al Fractionated endocardial electrograms are associated with slow conduction in humans: evidence from pace-mapping. J Am Coll Cardiol 1989;13:369–376. 10.1016/0735-1097(89)90514-7 2913115

[5] Hsia HH, Marchlinski FE. Electrophysiology studies in patients with dilated cardiomyopathies. Card Electrophysiol Rev 2002;6:472–481. 10.1023/a:1021109130276 12438831

